# Comparison of US Oncologist Rurality by Practice Setting and Patients Served

**DOI:** 10.1001/jamanetworkopen.2023.50504

**Published:** 2024-01-05

**Authors:** Sarah L. Cornelius, Andrew P. Shaefer, Sandra L. Wong, Erika L. Moen

**Affiliations:** 1 Department of Biomedical Data Science, Geisel School of Medicine at Dartmouth, Lebanon, New Hampshire; 2 The Dartmouth Institute for Health Policy and Clinical Practice, Lebanon, New Hampshire; 3 Department of Surgery, Geisel School of Medicine at Dartmouth, Lebanon, New Hampshire

## Abstract

**Importance:**

Studies of the oncology workforce most often classify physician rurality by their practice location, but this could miss the true extent of physicians involved in rural cancer care.

**Objective:**

To compare a method for identifying oncology physicians involved in rural cancer care that uses the proportion of rural patients served with the standard method based on practice location.

**Design, Setting, and Participants:**

This cross-sectional study used retrospective Centers for Medicare & Medicaid Services encounter data on medical oncologists, radiation oncologists, and surgeons treating Medicare beneficiaries diagnosed with breast, colorectal, or lung cancer from January 1 to December 31, 2019. Data were analyzed from May to September 2023.

**Main Outcomes and Measures:**

The standard method of classifying oncologist physician rurality based on practice location was compared with a novel method of classification based on proportion of rural patients served.

**Results:**

The study included 27 870 oncology physicians (71.3% male), of whom 835 (3.0%) practiced in a rural location. Physicians practicing in a rural location treated a high proportion of rural patients (median, 50.0% [IQR, 16.7%-100%]). When considering the rurality of physicians’ patient panels, 5123 physicians (18.4%) whose patient panel included at least 20% rural patients, 3199 (11.5%) with at least 33% rural patients, and 1996 (7.2%) with at least 50% rural patients were identified. Using a physician’s patient panel to classify physician rurality revealed a higher number and greater spread of oncology physicians involved in rural cancer care in the US than the standard method, while maintaining high performance (area under the curve, 0.857) and fair concordance (κ, 0.346; 95% CI, 0.323-0.369) with the method based on practice setting.

**Conclusions and Relevance:**

In this cross-sectional study, classifying oncologist rurality by the proportion of rural patients served identified more oncology physicians treating patients living in rural areas than the standard method of practice location and may more accurately capture the rural cancer physician workforce, as many hospitals have historically been located in more urban areas. This new method may be used to improve future studies of rural cancer care delivery.

## Key Points

**Question:**

How can oncology physicians providing care to patients living in rural areas be accurately identified?

**Findings:**

In this cross-sectional study of 27 870 oncology physicians, using physicians’ patient panels to classify physician rurality identified more oncology physicians treating patients living in rural areas than standard methods that rely on only practice location, while maintaining high performance and fair concordance between the 2 methods.

**Meaning:**

The patient panel classification method expanded on standard classification methods and may more accurately capture oncologists involved in rural cancer care.

## Teaser

This cross-sectional study assesses a method of classifying oncology physician rurality using proportion of rural patients served compared with a method based on practice location.

## Introduction

While rural physicians make up only a small portion of physicians in the US, they reach a substantial portion of the population.^1,2,3,4^ Only about 3% of oncologists practice in a rural setting,^5^ but workforce studies most often classify physician rurality by their practice location. This approach, given its high specificity, could miss the true extent of physicians involved in rural cancer care. Identifying the physicians involved in rural cancer care is critical for studying the challenges in care access and coordination that rural patients often face, including less access to routine health care,^6,7^ less access to specialized cancer care,^8,9,10^ and worse cancer survival and health outcomes.^11^ In this study, we aimed to develop a method for classifying physician rurality based on proportion of rural patients served, which could expand on methods using only practice setting.

## Methods

### Study Cohort

In this retrospective cross-sectional study, we used a 100% Centers for Medicare & Medicaid Services (CMS) file to identify beneficiaries with an incident diagnosis of breast (*International Statistical Classification of Diseases and Related Health Problems, Tenth Revision* [*ICD-10*] code C50.x), lung (*ICD-10* code C34.x), or colorectal (*ICD-10* codes C18.x, C19, and C20) cancer from January 1 to December 31, 2019, using an algorithm previously validated for Medicare claims data.^12^ Physicians caring for these patients were identified from CMS MedPAR and carrier files. Physician specialty was determined using the primary and first 2 subspecialty taxonomy codes in the National Plan and Provider Enumeration System, and we focused on medical oncologists, radiation oncologists, and surgeons who performed a cancer-related surgical procedure for cohort patients (eTable 1 in Supplement 1). This study followed the study design, analysis, and results reporting outlined by the Strengthening the Reporting of Observational Studies in Epidemiology (STROBE) reporting guideline for cross-sectional studies. The project was approved by the Dartmouth College Committee for the Protection of Human Subjects, which waived the need for informed consent as an exempt study because it was determined to be secondary research of existing data reported in a manner that individuals could not be identified.

### Rurality Classification

Rurality was assigned using Rural-Urban Commuting Area categorization B, which classifies codes as metropolitan, micropolitan, and rural (eTable 2 in Supplement 1).^13^ A 3-tier system was used for consistency with prior methods.^14^ Two methods were used to classify oncology physician rurality. The first method was based on a physician’s primary practice zip code, which was assigned based on the plurality of their encounters in the CMS carrier file. The second method involved a calculation of the percentage of patients who resided in a rural zip code who were seen by the physician. Patient rurality was determined from their residential zip codes in the Medicare Beneficiary Summary File. We then conducted a subanalysis to expand who was classified as an oncologist practicing in a rural location, which addressed physicians practicing at multiple sites. Rather than using plurality of care, oncologists were categorized as practicing in a rural location if they provided any care in a rural setting. Among those who did not have any encounters in a rural setting, plurality of care was used to distinguish between predominantly metropolitan and micropolitan physicians.

### Statistical Analysis

Data were analyzed from May to September 2023. We compared our new rurality classification method (ie, proportion of rural patients served) with the gold standard classification method (ie, practice location) using summary statistics, a receiver operating characteristic curve, and Cohen κ for multiple cutoff values. We summarized differences in characteristics between rural and urban oncology physicians using these methods for categorizing rurality. Location rurality was stratified into 3 bins: metropolitan, micropolitan, and rural. Rurality based on patient panel was stratified into no rural patients, low rural patient population (<20%), and 3 levels for high rural patient population (≥20%, ≥33%, and ≥50%). Maps illustrated geographic differences in the distribution of the rural oncologist workforce using these 2 classification methods in the US. Analyses were performed using RStudio, version 4.3.1 (R Core Team).

## Results

**Figure 1.**
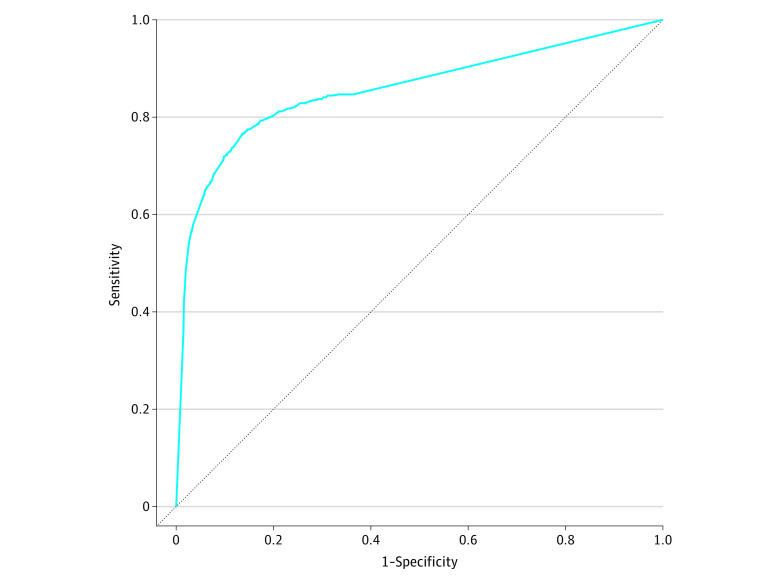
Comparison of Classifying Oncologist Physician Rurality Based on Practice Location vs Proportion of Rural Patients Served

The cohort included 27 870 oncology physicians, of whom 835 (3.0%) practiced in a rural location. Of the total physicians, 7989 (28.7%) were female and 19 881 (71.3%) were male. The majority of physicians saw no or few rural patients (median, 0% [IQR, 0%-12.5%]). Physicians practicing in a rural location treated a higher proportion of rural patients (median, 50.0% [IQR, 16.7%-100%]) compared with their urban counterparts (median, 0% [IQR, 0%-10.9%]). We identified 5123 oncology physicians (18.4%) whose patient panel included at least 20% rural patients, 3199 (11.5%) with at least 33% rural patients, and 1996 (7.2%) with at least 50% rural patients. While using the patient panel classified more physicians as rural than the practice location method, the 2 classification methods performed consistently (area under the curve, 0.857) (Figure 1). We found that a 2% rural patient panel cutoff maximized both sensitivity and specificity when comparing a physician’s rural patient panel with their practice location but resulted in low concordance (κ, 0.077; 95% CI, 0.071-0.083) (Table 1). We prioritized specificity when identifying relevant cutoff values. Among physicians who treated any rural patients, the patient panel consisted of a median of 16.7% rural patients (IQR, 8.1%-33.3%). Additionally, the 75th and 85th percentiles were 33% and 50%, respectively. The cutoffs of 20%, 33%, and 50% corresponded to a specificity of 0.981 (95% CI, 0.979-0.982), 0.984 (95% CI, 0.983-0.986), and 0.999 (95% CI, 0.999-1.00), respectively. These cutoffs resulted in slight (κ, 0.177; 95% CI, 0.165-0.190 [20% cutoff]) to fair (κ, 0.259; 95% CI, 0.241-0.277 [33% cutoff] and 0.346; 95% CI, 0.323-0.369 [50% cutoff]) concordance with physician location.

**Table 1.** Concordance Between Classifying Oncologist Physician Rurality Based on Practice Location vs Proportion of Rural Patients Served

Proportion of rural patients served by physicians, %	Cohen κ (95% CI)
2	0.077 (0.071-0.083)
10	0.117 (0.108-0.126)
20	0.177 (0.165-0.190)
33	0.259 (0.241-0.277)
40	0.310 (0.289-0.331)
50	0.346 (0.323-0.369)
60	0.426 (0.399-0.453)
80	0.425 (0.395-0.456)
100	0.375 (0.343-0.406)

The characteristics of rural and urban physicians using both classification methods were similar. Oncology physicians practicing in a rural location (practice location: n = 835; ≥50% patient population: n = 1996) were less likely to treat lung cancer (location: 453 [54.3%]; patient population: 1127 [56.5%]) compared with breast (location: 730 [87.4%]; patient population: 1583 [79.3%]) or colorectal (location: 749 [89.7%]; patient population: 1692 [84.8%]) cancer (Table 2). We observed a higher proportion of male physicians than female physicians in rural settings (location: 677 [81.1%] vs 158 [18.9%]; patient population: 1587 [79.5%] vs 409 [20.5%]). We found an inverse relationship with patient volume when classifying rurality based on practice location, in which mean (SD) patient volume steadily decreased with increasing rurality (urban: 13.6 [15.8]; rural: 7.9 [9.7]). Conversely, we found that physicians with no rural patients had a lower mean patient volume than those who saw rural patients. Physicians with a low proportion of rural patients provided care to 180% more patients than those who treated no rural patients (mean [SD] patient volume: 26.6 [20.1] vs 9.5 [11.7]). These observations were consistent after stratifying by specialist type (eTables 3-5 in Supplement 1) and when incorporating oncologists practicing at a secondary rural site (eTable 6 in Supplement 1).

**Table 2.** Characteristics of Rural and Urban Oncologists Using Methods of Classifying Oncologist Physician Rurality Based on Practice Location vs Proportion of Rural Patients Served^a^

Characteristic	Practice location			Proportion of rural patients served				
	Metropolitan (n = 24 475)	Micropolitan (n = 2560)	Rural (n = 835)	0% (n = 17 331)	<20% (n = 5416)	≥20% (n = 5123)^b^	≥33% (n = 3199)^b^	≥50% (n = 1996)^b^
Cancer type^c^								
Breast	19 375 (79.2)	2311 (90.3)	730 (87.4)	13 225 (76.3)	4955 (91.5)	4236 (82.7)	2596 (81.2)	1583 (79.3)
Lung	15 541 (63.5)	1742 (68.0)	453 (54.3)	10 065 (58.1)	4227 (78.0)	3444 (67.2)	2011 (62.9)	1127 (56.5)
Colorectal	19 073 (77.9)	2327 (90.9)	749 (89.7)	13 334 (76.9)	4460 (82.3)	4355 (85.0)	2725 (85.2)	1692 (84.8)
Sex								
Female	7295 (29.8)	536 (20.9)	158 (18.9)	4970 (28.7)	1830 (33.8)	1189 (23.2)	686 (21.4)	409 (20.5)
Male	17 180 (70.2)	2024 (79.1)	677 (81.1)	12 361 (71.3)	3586 (66.2)	3934 (76.8)	2513 (78.6)	1587 (79.5)
Specialty								
Medical oncologist	9433 (38.5)	800 (31.3)	202 (24.2)	6493 (37.5)	2295 (42.4)	1647 (32.1)	938 (29.3)	522 (26.2)
Radiation oncologist	3843 (15.7)	316 (12.3)	74 (8.9)	2186 (12.6)	1315 (24.3)	732 (14.3)	356 (11.1)	154 (7.7)
Surgical oncologist	11 199 (45.8)	1444 (56.4)	559 (66.9)	8652 (49.9)	1806 (33.3)	2744 (53.6)	1905 (59.5)	1320 (66.1)
Practice								
≥10 y Experience^d^	21 129 (86.3)	2218 (86.6)	721 (86.3)	14 971 (86.4)	4764 (88.0)	4333 (84.6)	2676 (83.7)	1665 (83.4)
Patient volume, mean (SD)	13.6 (15.8)	12.3 (13.3)	7.9 (9.7)	9.5 (11.7)	26.6 (20.1)	12.3 (13.5)	9.31 (11.1)	6.6 (8.9)

^a^ Data are presented as number (percentage) of physicians unless otherwise indicated.

^b^ These groups are not mutually exclusive.

^c^ Oncologists could treat more than 1 cancer type.

^d^ Calculated as the number of years between National Provider Identifier enumeration date and January 1, 2019.

**Figure 2.**
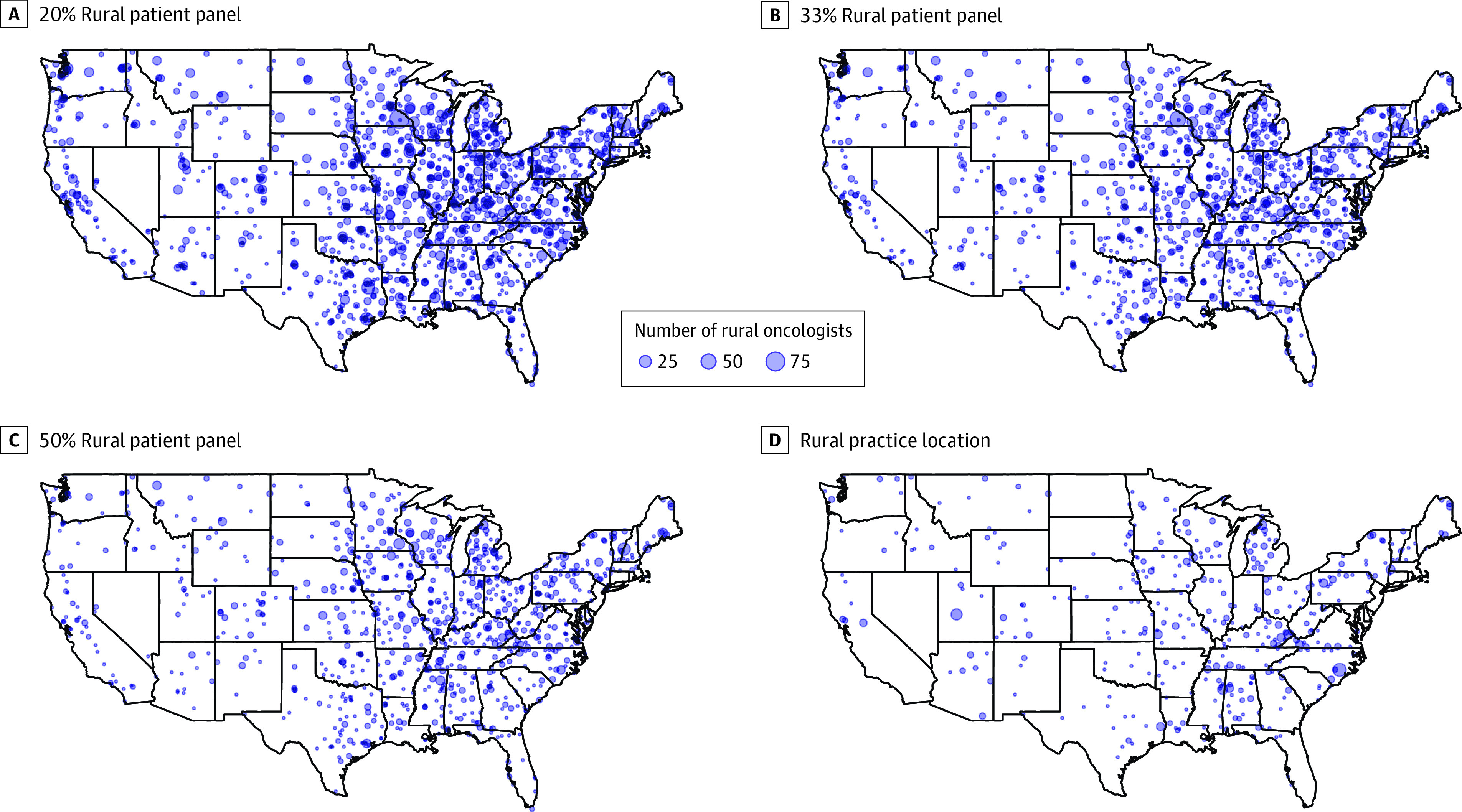
Distribution of Rural Oncologists in the US Using Methods of Classifying Oncologist Physician Rurality Based on Practice Location vs Proportion of Rural Patients Served

Among oncology physicians practicing in a rural location, 529 of 835 (63.4%) had a patient panel of at least 50% (Table 3). Conversely, only 989 of 24 475 physicians (4.0%) practicing in a metropolitan location had a rural patient panel of at least 50%. Classifying oncologist rurality by their patient panel identified new areas across the US with rural providers (Figure 2), including in southern California, Maine, North Dakota, and Montana.

**Table 3.** Comparison of the Counts of Rural Oncologists Using Practice Location and Rural Patient Panel

Rural patients, %	Physicians, by location, No. (%)		
	Metropolitan (n = 24 475)	Micropolitan (n = 2560)	Rural (n = 835)
0	16 284 (66.5)	919 (35.9)	128 (15.3)
<20	4848 (19.8)	516 (20.2)	52 (6.2)
≥20^a^	3343 (13.7)	1125 (43.9)	655 (78.4)
≥33^a^	1834 (7.5)	771 (30.1)	594 (71.1)
≥50^a^	989 (4.0)	478 (18.7)	529 (63.4)

^a^ These groups are not mutually exclusive.

## Discussion

Rural patients with cancer can face unique challenges at all stages of their cancer journey from diagnosis to treatment to survivorship. Not only are rural patients affected by social and demographic contributors to cancer, which can lead to increased cancer risks and reduced survivorship,^15^ these patients can also experience significant geographic disparities compared with their urban counterparts.^8,10^ There is a critical need to correctly identify which physicians are providing rural cancer care to better understand the true effects of rurality on disparities in care access and quality.

The characteristics of oncologists were consistent between the 2 rurality classification methods, but we identified differences in some characteristics between predominantly rural and predominantly metropolitan oncologists. Interestingly, we found that regardless of our classification method, predominantly rural oncologists were less likely to treat lung cancer, be a radiation oncologist, and be female. While uneven geographic distribution of oncology specialists has been established, more needs to be done to understand disparities for specific cancer types and oncology specialties.^10,16,17^ These rural disparities may be influenced by fewer training programs in rural areas, debt and salary concerns, or simply by limited access to specialized equipment to conduct complex therapy.^10,18^

In this study, we identified a novel method for classifying physician rurality based on their patient panel that expanded on current methods using practice location. Use of the new method revealed a higher number and greater spread of oncology physicians involved in rural cancer care in the US while having high performance and fair concordance with practice location. The method also provided a continuous measure of physician rurality and 4 relevant cutoff values with varying levels of sensitivity and specificity that may be used for future studies of rural cancer care. Future work should also incorporate the use of telehealth when categorizing a physician’s rurality. While rare prior to 2020, telehealth use in cancer care increased substantially during the COVID-19 pandemic, and its use may extend the cohort of physicians involved in rural cancer care delivery.^19^

### Limitations

There are limitations to the proposed method. First, our method is most suitable for studies with access to patient utilization data, as patient panel rurality is not readily available from other data sources. Second, we assigned physicians to a primary practice location based on the plurality of their care, but some physicians travel between urban and rural hospitals. This could have led to underestimation of the volume of rural physicians. Third, we may have underestimated the number of oncology physicians in the cohort. Taxonomy codes from the National Plan and Provider Enumeration System, while fairly specific, can be misassigned, or there may be delays in updating when specialties are added. Fourth, we focused solely on medical, radiation, and surgical oncologists for this study. A cancer team consists of more specialties, such as physician assistants and nurse practitioners, who are often integral to the delivery of rural cancer care; however, the care they provide is difficult to capture using Medicare claims. Future studies may need to investigate methods to identify these crucial cancer practitioners.

## Conclusions

In this study, even at our most restrictive cutoff, our new rurality classification method using proportion of rural patients served identified 139% more oncology physicians involved in rural cancer care across the US than the standard method based on practice location. We used CMS claims and focused on oncology care for this study, but these methods are likely generalizable to other data sources and medical indications. While studies of care access may benefit from using practice location to define rurality, patient panels may better inform questions about the physicians involved in delivering cancer care to rural patients. Future studies should carefully consider how physician rurality is classified to accurately represent who is involved in providing rural cancer care.

## Notes

Supplement 1. **eTable 1.** Taxonomy Codes Used to Classify Oncologist Specialty **eTable 2.** Rurality Categorization Based on Rural-Urban Commuting Area (RUCA) Codes **eTable 3.** Characteristics of Rural and Urban Medical Oncologists Using Two Rurality Classification Methods **eTable 4.** Characteristics of Rural and Urban Radiation Oncologists Using Two Rurality Classification Methods **eTable 5.** Characteristics of Rural and Urban Surgical Oncologists Using Two Rurality Classification Methods **eTable 6.** Characteristics of Rural and Urban Oncologists Incorporating Physicians Practicing at Multiple Sites

Supplement 2.
Data Sharing Statement
