# Supplementary material for: Comparison of US Oncologist Rurality by Practice Setting and Patients Served
Source: JAMA Netw Open. 2024 Jan 5;7(1):e2350504. doi: 10.1001/jamanetworkopen.2023.50504 (PMC10770776; doi:10.1001/jamanetworkopen.2023.50504)
Supplement: Supplement 1. — eTable 1. Taxonomy Codes Used to Classify Oncologist Specialty eTable 2. Rurality Categorization Based on Rural-Urban Commuting Area (RUCA) Codes eTable 3. Characteristics of Rural and Urban Medical Oncologists Using Two Rurality Classification Methods eTable 4. Characteristics of Rural and Urban Radiation Oncologists Using Two Rurality Classification Methods eTable 5. Characteristics of Rural and Urban Surgical Oncologists Using Two Rurality Classification Methods eTable 6. Characteristics of Rural and Urban Oncologists Incorporating Physicians Practicing at Multiple Sites [file jamanetwopen-e2350504-s001.pdf]

Supplemental Online Content

Cornelius SL, Shaefer AP, Wong SL, Moen EL. Comparison of US oncologist rurality by practice setting and patients served. *JAMA Netw Open.* 2024;7(1):e2350504. doi:10.1001/jamanetworkopen.2023.50504

eTable 1. Taxonomy Codes Used to Classify Oncologist Specialty

eTable 2. Rurality Categorization Based on Rural-Urban Commuting Area (RUCA) Codes

eTable 3. Characteristics of Rural and Urban Medical Oncologists Using Two Rurality Classification Methods

eTable 4. Characteristics of Rural and Urban Radiation Oncologists Using Two Rurality Classification Methods

eTable 5. Characteristics of Rural and Urban Surgical Oncologists Using Two Rurality Classification Methods

eTable 6. Characteristics of Rural and Urban Oncologists Incorporating Physicians Practicing at Multiple Sites

This supplemental material has been provided by the authors to give readers additional information about their work.

eTable 1. Taxonomy codes used to classify oncologist specialty.	
**Specialty**	**Taxonomy Code**
Medical oncologist	207RH0003X, 207RX0202X, 207VX0201X
Radiation oncologist	2085R0001X
Surgeon	2086X0206X, 208600000X, 2086S0122X, 208200000X, 208G00000X, 208C00000X

© 2024 Cornelius SL et al. *JAMA Network Open.*

eTable 2. Rurality categorization based on Rural-Urban Commuting Area (RUCA) codes.

Rurality Categorization	RUCA Code
Metropolitan	1.0, 1.1, 2.0, 2.1, 3.0, 4.1, 5.1, 7.1, 8.1, and 10.1
Micropolitan	4.0, 4.2, 5.0, 5.2, 6.0, and 6.1
Rural	7.0, 7.2, 7.3, 7.4, 8.0, 8.2, 8.3, 8.4, 9.0, 9.1, 9.2, 10.0, 10.2, 10.3, 10.4, 10.5, and 10.6

© 2024 Cornelius SL et al. *JAMA Network Open.*

eTable 3. Characteristics of rural and urban medical oncologists using methods of classifying oncologist physician rurality based on practice location vs proportion of rural patients served[a]

	Practice location			Proportion of rural patients served				
Characteristic	Metropolitan (N=9,433)	Micropolitan (N=800)	Rural (N=202)	0% (N=6,493)	<20% (N=2,295)	≥20%[b] (N=1,647)	≥33%[b] (N=938)	≥50%[b] (N=522)
**Cancer type[c]**								
Breast	8,503 (90.1)	777 (97.1)	196 (97.0)	5,671 (87.3)	2,249 (98.0)	1,556 (94.5)	874 (93.2)	469 (89.8)
Lung	7,957 (84.4)	746 (93.3)	188 (93.1)	5,222 (80.4)	2,186 (95.3)	1,483 (90.0)	818 (87.2)	425 (81.4)
Colorectal	8,078 (85.6)	757 (94.6)	188 (93.1)	5,387 (83.0)	2,139 (93.2)	1,497 (90.9)	844 (90.0)	448 (85.8)
**Sex**								
Female	3,346 (35.5)	202 (25.3)	54 (26.7)	2,301 (35.4)	820 (35.7)	481 (29.2)	274 (29.2)	162 (31.0)
Male	6,087 (64.5)	598 (74.8)	148 (73.3)	4,192 (64.6)	1,475 (64.3)	1,166 (70.8)	664 (70.8)	360 (69.0)
**Practice**								
≥10 years experience[d]	8,192 (86.8)	708 (88.5)	173 (85.6)	5,638 (86.8)	2,037 (88.8)	1,398 (84.9)	774 (82.5)	418 (80.1)
Patient volume, M (SD)	15.0 (16.6)	18.4 (14.6)	13.8 (11.4)	10.7 (11.7)	27.7 (21.8)	15.8 (14.2)	13.1 (12.7)	9.88 (11.3)

[a]Data are presented as number (percentage) of physicians unless otherwise indicated.
[b]These groups are not mutually exclusive.
[c]Oncologists could treat more than 1 cancer type.
[d]Calculated as the number of years between National Provider Identifier enumeration date and January 1, 2019.

© 2024 Cornelius SL et al. *JAMA Network Open.*

eTable 4. Characteristics of rural and urban radiation oncologists using methods of classifying oncologist physician rurality based on practice location vs proportion of rural patients served[a]

	Practice location			Proportion of rural patients served				
Characteristic	Metropolitan (N=3,843)	Micropolitan (N=316)	Rural (N=74)	0% (N=2,186)	<20% (N=1,315)	≥20%[b] (N=732)	≥33%[b] (N=356)	≥50%[b] (N=154)
**Cancer type[c]**								
Breast	3,742 (97.4)	316 (100)	73 (98.6)	2,103 (96.2)	1,304 (99.2)	724 (98.9)	353 (99.2)	153 (99.4)
Lung	3,636 (94.6)	310 (98.1)	69 (93.2)	2,014 (92.1)	1,289 (98.0)	712 (97.3)	343 (96.3)	147 (95.5)
Colorectal	3,276 (85.2)	296 (93.7)	59 (79.7)	1,755 (80.3)	1,209 (91.9)	667 (91.1)	314 (88.2)	131 (85.1)
**Sex**								
Female	1,054 (27.4)	55 (17.4)	10 (13.5)	620 (28.4)	354 (26.9)	145 (19.8)	70 (19.7)	24 (15.6)
Male	2,789 (72.6)	261 (82.6)	64 (86.5)	1,566 (71.6)	961 (73.1)	587 (80.2)	286 (80.3)	130 (84.4)
**Practice**								
≥10 years experience[d]	3,050 (79.4)	286 (90.5)	65 (87.8)	1,734 (79.3)	1,071 (81.4)	596 (81.4)	297 (83.4)	129 (83.8)
Patient volume, M (SD)	23.2 (18.8)	22.5 (16.9)	17.2 (15.1)	17.4 (15.8)	32.8 (19.7)	22.2 (17.0)	18.9 (15.6)	15.9 (15.2)

[a]Data are presented as number (percentage) of physicians unless otherwise indicated.
[b]These groups are not mutually exclusive.
[c]Oncologists could treat more than 1 cancer type.
[d]Calculated as the number of years between National Provider Identifier enumeration date and January 1, 2019.

© 2024 Cornelius SL et al. *JAMA Network Open.*

eTable 5. Characteristics of rural and urban surgical oncologists using methods of classifying oncologist physician rurality based on practice location vs proportion of rural patients served[a]

	Practice location			Proportion of rural patients served				
Characteristic	Metropolitan (N=11,199)	Micropolitan (N=1,444)	Rural (N=559)	0% (N=8,652)	<20% (N=1,806)	≥20%[b] (N=2,744)	≥33%[b] (N=1,905)	≥50%[b] (N=1,320)
**Cancer type[c]**								
Breast	7,130 (63.7)	1,218 (84.3)	461 (82.5)	5,451 (63.0)	1,402 (77.6)	1,956 (71.3)	1,369 (71.9)	961 (72.8)
Lung	3,948 (35.3)	686 (47.5)	196 (35.1)	2,829 (32.7)	752 (41.6)	1,249 (45.5)	850 (44.6)	555 (42.0)
Colorectal	7,719 (68.9)	1,274 (88.2)	502 (89.8)	6,192 (71.6)	1,112 (61.6)	2,191 (79.8)	1,567 (82.3)	1,113 (84.3)
**Sex**								
Female	2,895 (25.9)	279 (19.3)	94 (16.8)	2,049 (23.7)	656 (36.3)	563 (20.5)	342 (18.0)	223 (16.9)
Male	8,304 (74.1)	1,165 (80.7)	465 (83.2)	6,603 (76.3)	1,150 (63.7)	2,181 (79.5)	1,563 (82.0)	1,097 (83.1)
**Practice**								
≥10 years experience[d]	9,887 (88.3)	1,224 (84.8)	483 (86.4)	7,599 (87.8)	1,656 (91.7)	2,339 (85.2)	1,605 (84.3)	1,118 (84.7)
Patient volume, M (SD)	9.18 (12.0)	6.63 (7.66)	4.56 (5.19)	6.61 (9.07)	20.7 (16.1)	7.45 (9.34)	5.63 (6.65)	4.20 (4.79)

[a]Data are presented as number (percentage) of physicians unless otherwise indicated.
[b]These groups are not mutually exclusive.
[c]Oncologists could treat more than 1 cancer type.
[d]Calculated as the number of years between National Provider Identifier enumeration date and January 1, 2019.

© 2024 Cornelius SL et al. *JAMA Network Open.*

eTable 6. Characteristics of rural and urban oncologists incorporating physicians practicing at multiple sites[a]			
	**Practice location**		
Characteristic	Metropolitan (N=23,935)	Micropolitan (N=2,391)	Rural[b] (N=1,544)
**Cancer type[c]**			
Breast	18,899 (79.0)	2,158 (90.3)	1,359 (88.0)
Lung	15,159 (63.3)	1,630 (68.2)	947 (61.3)
Colorectal	18,604 (77.7)	2,167 (90.6)	1,378 (89.2)
**Sex**			
Female	7,172 (30.0)	508 (21.2)	309 (20.0)
Male	16,763 (70.0)	1,883 (78.8)	1,235 (80.0)
**Specialty**			
Medical oncologist	9,196 (38.4)	741 (31.0)	498 (32.3)
Radiation oncologist	3,766 (15.7)	295 (12.3)	172 (11.1)
Surgical oncologist	10,973 (45.8)	1,355 (56.7)	874 (56.6)
**Practice**			
≥10 years experience[d]	20,659 (86.3)	2,069 (86.5)	1,340 (86.8)
Patient volume, M (SD)	13.6 (15.8)	12.1 (12.6)	11.2 (13.8)

[a]Data are presented as number (percentage) of physicians unless otherwise indicated.
[b]Oncologists were categorized as rural if they had any clinical encounters in a rural ZIP Code; otherwise, the location where they provided the plurality of their care was used.
[c]Oncologists could treat more than 1 cancer type
[d]Calculated as the number of years between NPI enumeration date and January 1, 2019.

© 2024 Cornelius SL et al. *JAMA Network Open.*
