# Supplementary material for: Comparison of US Oncologist Rurality by Practice Setting and Patients Served
Source: JAMA Netw Open. 2024 Jan 5;7(1):e2350504. doi: 10.1001/jamanetworkopen.2023.50504 (PMC10770776; doi:10.1001/jamanetworkopen.2023.50504)
Supplement: Supplement 2. — Data Sharing Statement [file jamanetwopen-e2350504-s002.pdf]

Data Sharing Statement

Cornelius. Comparison of US Oncologist Rurality by Practice Setting and Patients Served.
JAMA Netw Open. Published January 05, 2024. doi:10.1001/jamanetworkopen.2023.50504

Data
Data available: No

Additional Information
Explanation for why data not available: As per our Data Use Agreement with CMS, we are not able to share data with individuals not approved by our DUA.
